# Cholesterol oxidase from *Rhodococcus erythropolis* with high specificity toward β-cholestanol and pytosterols

**DOI:** 10.1371/journal.pone.0241126

**Published:** 2020-10-26

**Authors:** Noriyuki Doukyu, Makoto Ishikawa

**Affiliations:** 1 Department of Life Sciences, Toyo University, Itakura-machi, Gunma, Japan; 2 Graduate School of Life Sciences, Toyo University, Itakura-machi, Gunma, Japan; 3 Bio-Nano Electronic Research Center, Toyo University, Kawagoe, Saitama, Japan; Universidade Nova de Lisboa Instituto de Tecnologia Quimica e Biologica, PORTUGAL

## Abstract

Two genes (*choRI* and *choRII*) encoding cholesterol oxidases belonging to the vanillyl-alcohol oxidase (VAO) family were cloned on the basis of putative cholesterol oxidase gene sequences in the genome sequence data of *Rhodococcus erythropolis* PR4. The genes corresponding to the mature enzymes were cloned in a pET vector and expressed in *Escherichia coli*. The two cholesterol oxidases produced from the recombinant *E*. *coli* were purified to examine their properties. The amino acid sequence of ChoRI showed significant similarity (57%) to that of ChoRII. ChoRII was more stable than ChoRI in terms of pH and thermal stability. The substrate specificities of these enzymes differed distinctively from one another. Interestingly, the activities of ChoRII toward β-cholestanol, β-sitosterol, and stigmasterol were 2.4-, 2.1-, and 1.7-fold higher, respectively, than those of cholesterol. No cholesterol oxidases with high activity toward these sterols have been reported so far. The cholesterol oxidation products from these two enzymes also differed. ChoRI and ChoRII oxidized cholesterol to form cholest-4-en-3-one and 6β-hydroperoxycholest-4-en-3-one, respectively.

## Introduction

An essential component of mammalian cells, cholesterol is involved in cell function and viability. It is a principal component of the plasma membrane and is a precursor to steroid hormones, bile acids, and vitamin D. However, high serum cholesterol levels are associated with increased risk for cardiovascular disease and many cancers [1]. Cholesterol oxidases (EC 1.1.3.6) have been widely used for the clinical determination of serum cholesterol level [2]. Cholesterol oxidases contain a flavin adenine dinucleotide (FAD) as the redox cofactor and generally oxidize cholesterol (cholest-5-en-3β-ol) to form cholest-4-en-3-one (CEO) with the reduction of oxygen to hydrogen peroxide [3, 4]. In addition, cholesterol oxidases have varieties of applications, including the production of valuable steroidal compounds [5, 6] and nonsteroidal chiral compounds [7] as well as insecticidal activity [8, 9]. Cholesterol oxidase from *Chromobacterium* sp. DS-1 has been shown to reduce oxysterol cytotoxicity in human fibroblasts [10].

A variety of bacteria are known to produce cholesterol oxidases that are involved in the first step of cholesterol degradation [11]. Cholesterol oxidases of pathogenic bacteria act as a virulence factor in pathogenicity [12]. In addition, *Streptomyces natalensis* produces a cholesterol oxidase that serves as a signaling protein for the biosynthesis of an antifungal antibiotic, polyene macrolide pimaricin [13]. It has also been reported that Alzheimer’s disease β-amyloid selectively oxidizes cholesterol to form cholest-4-en-3-one and therefore mimics the activity of cholesterol oxidase [14].

Based on the structures, cholesterol oxidases have been classified into two distinct protein families: a glucose/methanol/choline (GMC) oxidoreductase family and a vanillyl-alcohol oxidase (VAO) family. Cholesterol oxidases classified as the GMC oxidoreductase family have been found mostly in actinomycetes such as *Streptomyces* spp., *Rhodococcus* spp., and *Brevibacterium sterolicum* [15]. On the other hand, cholesterol oxidases belonging to the VAO family have been found not only in several actinomycetes but also in beta- and gamma-proteobacteria such as *Burkholderia cepacia* ST-200 [16, 17], *Pseudomonas aeruginosa* strain PA157 [18], and *Chromobacterium* sp. DS-1 [15, 19].

Although cholesterol oxidases generally catalyze the oxidation of cholesterol to form CEO, several cholesterol oxidases belonging to the VAO family from *B*. *cepacia* ST-200, *Chromobacterium* sp. DS-1, and *P*. *aeruginosa* strain PA157 oxidize cholesterol to 6β-hydroperoxycholest-4-en-3-one (HCEO) [18–20]. These enzymes possess relatively higher stability against high temperatures, organic solvents, and detergents than commercially available cholesterol oxidases.

*R*. *erythropolis* PR4 has been isolated from the deep sea south of Okinawa Island, Japan [21]. Strain PR4 can assimilate *n*-alkanes, alkylbenzenes, and pristine [22]. It can also produce large amounts of extracellular polysaccharides, which are assumed to play important roles in tolerance to a variety of organic solvents [23, 24]. On the basis of the putative cholesterol oxidase gene sequence in the genome sequence data of strain PR4, we identified two cholesterol oxidases belonging to the VAO family. In this study, we report the cloning of both cholesterol oxidase genes from *R*. *erythropolis* PR4, the expression of the genes in *E*. *coli*, and a comparative study on the characteristics of these two enzymes.

## Materials and methods

### Strains, media, and plasmids

*E*. *coli* DH5α [*supE44*, Δ*lac*U169(ϕ80*lacZ*Δ*M15*), *hsdR17*, *recA1*, *endA1*, *gyrA96*, *thi-1*, *relA1*) and *E*. *coli* Rosetta (DE3) pLysS [F^-^, *omp*T, *hsd*S_B_(R_B_^-^ m_B_^-^), *gal*, *dcm*, *lacYI* (DE3), pLysSRARE (Cam^R^)] (Merck Millipore, Burlington, MA, USA) were used as hosts for the recombinant plasmids. pET-21b(+) was obtained also from Merck Millipore. *E*. *coli* strains were grown in Luria–Bertani broth (LB medium). When necessary, the medium was solidified with 1.5% (w/v) agar and supplemented with appropriate antibiotics. The recombinant *E*. *coli* Rosetta strain was grown in LB+AC medium, which has the same composition as LB medium except that the medium was supplemented with 50 μg/ml ampicillin and 25 μg/ml chloramphenicol.

### Cloning of cholesterol oxidase genes and construction of expression plasmids

Two putative cholesterol oxidase gene sequences (locus tag RER_07350 and RER_04530) of *R*. *erythropolis* PR4 determined via a genome sequence analysis were deposited in the National Center for Biotechnology Information (NCBI) database under accession number AP008957. Cholesterol oxidase genes corresponding to locus tags of RER_07350 and RER_04530 were named *choRI* and *choRII* in this study, respectively. PCR primers were designed based on the sequence of *R*. *erythropolis* PR4. PCR was performed to amplify the fragment, including the entire cholesterol oxidase gene, using PrimeSTAR HS DNA polymerase (Takara Bio) and genomic DNA of strain PR4 as the template. The primers for *choRI* were ChoRI-S, 5’-AAACATATGGTCCCCGCCGGCTCCTCCGG-3’ (NdeI site underlined) and ChoRI-AS, 5’-TTTGCGGCCGCGGGCAGCAACTGGTCCAGGAA-3’ (NotI site underlined). On the other hand, the primers for *choRII* were ChoRII-S, 5’-AAACATATGGTTCCCTGGGGCTCGGCATC-3’ (NdeI site underlined) and ChoRII-AS, 5’-TTTGCGGCCGCGCTGGGCGGCAAGACGCGGTC-3’ (NotI site underlined). Both sense primers were designed to incorporate the restriction enzyme site NdeI and to remove the signal peptide. In addition, both anti-sense primers were designed to contain the C-terminal 6 × His-Tag. The thermal cycling parameters were 94°C for 1 min followed by 30 cycles of 94°C for 1 min, 50°C for 1 min, and 72°C for 2 min. The obtained PCR fragments digested with the restriction enzymes (NdeI and NotI) were cloned into the same sites in pET-21b(+). The resulting plasmids containing *choRI* and *choRII* were called pETchoRI and pETchoRII, respectively. It was confirmed by resequencing that there was no mutation in the cholesterol oxidase genes on these plasmids.

### Production and purification of the recombinant cholesterol oxidases

The *E*. *coli* Rosetta strain harboring pETchoRI or pETchoRII was grown in 200 ml of LB+AC medium at 30°C. When the culture reached an OD_660_ of approximately 0.5, protein expression was induced by adding 0.5 mM isopropyl-β-D-thiogalactopyranoside (IPTG). After incubation for another 18 h at 25°C, the cells were harvested by centrifugation (6,000 x g, 15 min, 4°C). The cell pellet was resuspended with 10 ml of 10 mM Tris-HCl (pH8.0) and disrupted by using the ultrasonic disruptor UD-200 (Tomy Seiko, Tokyo, Japan). Cell debris was removed by centrifugation (10,000 x g, 10 min, 4°C). The supernatant was loaded on a column (1.0 by 4 cm) of Ni Sepharose 6 Fast Flow (GE Healthcare, Chicago, IL, USA) equilibrated with a binding buffer (50 mM Tris-HCl, 500 mM NaCl, and 20 mM imidazole, pH 7.5). The recombinant enzyme was eluted with 10 ml of an elution buffer (50 mM Tris-HCl, 500 mM NaCl, and 500 mM imidazole, pH 7.5). The fractions with enzyme activity were pooled. This solution was dialyzed against 10 mM Tris-HCl buffer (pH 8.0) at 4°C and put on a column (2.5 by 6 cm) of DEAE-cellulose DE52 (Whatman, Maidstone, UK) equilibrated with the Tris-HCl buffer (pH 8.0). The column was washed with 50 ml of the Tris-HCl buffer (pH 8.0) and then eluted with a linear gradient of NaCl concentrations of 0 to 400 mM in 400 ml of the Tris buffer (pH 8.0). The enzyme solution was dialyzed against 10 mM Tris-HCl (pH8.0) at 4°C and kept at 4°C until used.

### Determination of protein concentrations

Protein concentrations were determined using the method of Bradford [25] and bovine serum albumin as the standard.

### Cholesterol oxidase activity assay and steady-state kinetics

Cholesterol oxidase activity was assayed by measuring the H_2_O_2_ generation accompanying the oxidation of cholesterol as described previously [16]. Assay solution consisted of 50 mM sodiumphosphate buffer (pH 7.0), 64 mM sodium cholate, 0.34% Triton X-100, 1.4 mM 4-aminoantipyrine, 21 mM phenol, 5 units of horseradish peroxidase (Toyobo Co., Ltd., Tsuruga, Japan), and 0.89 mM of cholesterol. The reaction was initiated with the addition of an appropriate amount of enzyme to the assay solution. The red color development was monitored at 500 nm. One unit of enzymatic activity was defined as the amount required to oxidize 1 μmol cholesterol/min at 30°C. Unless otherwise stated, this is the method we used to determine enzyme activity in this study. To investigate the optimum pH for enzyme activity, we estimated the cholesterol oxidase activity by monitoring the consumption of oxygen [19]. Assay solution consisted of 64 mM sodium cholate, 0.34% Triton X-100 and 0.89 mM of cholesterol and 100 mM of each buffer solution. Initial velocity of oxygen consumption was measured with an oxygen monitor at 30°C. To investigate the optimum temperature for enzyme activity, we estimated the cholesterol oxidase activity by measuring the A_249_ based on the λmax value of oxidized product, as described previously [19]. Assay solution consisted of 50 mM Tris-HCl buffer (pH 8.0), 64 mM sodium cholate, 5% isopropanol and 0.25 mM of cholesterol. The reaction was started by adding an appropriate amount of enzyme to the assay solution. After 5 min of incubation at 30°C, 2 ml of ethanol was added to the assay mixture to stop the reaction, and the formation of oxidized product was determined by measuring the A_249_.

To investigate the steady-state kinetics, cholesterol oxidation activity was examined by measuring H_2_O_2_ generation with assay solution containing 0 to 1 mM cholesterol. The kinetic parameters (Km and Vmax) were calculated by nonlinear regression using GraphPad Prism8 (GraphPad Software, Inc., CA, USA). The mean values and standard deviations of the Km and Vmax values were obtained from three independent experiments.

### Cholesterol oxidase stability assay

To examine the pH stability, cholesterol oxidase solution (0.2 U ml^-1^ in each buffer system) was determined after incubation at 30°C for 1 h under various pHs. The residual activity was examined by measuring H_2_O_2_ generation at 30°C. The buffer systems (100 mM) were glycine-HCl (pH 3.0), citrate-sodium citrate (pH 3.0–4.0), CH_3_COOH-CH_3_COONa (pH 5.0–5.5), NaH_2_PO_4_-Na_2_HPO_4_ (pH 5.5–7.5), Tris-HCl (pH 7.5–9.0), Na_2_CO_3_-NaHCO_3_ (pH 9.0–11.0), and NaCl-NaOH (pH 11.0–12.0).

To examine the enzymatic stability by the addition of detergents, cholesterol oxidase solution (0.2 U ml^-1^ in 50 mM sodium phosphate [pH 7.0]) containing 0.5% of each detergent was incubated at 30°C for 1 h. To investigate the stability in the presence of organic solvents, each organic solvent (1 ml) was added to 2 ml of the cholesterol oxidase solution described above. The mixture was incubated at 37°C with shaking for 24 h. In both cases, the residual activity was assayed by measuring the formation of H_2_O_2_.

### Isolation of the cholesterol oxidation product

Cholesterol oxidation products of ChoRI and ChoRII were isolated in a manner similar to that described previously [19]. Purified cholesterol oxidase (about 1 unit) was incubated in 100 ml of reaction mixture consisting of 50 mM phosphate buffer, pH 7.0, 64 mM sodium cholate, 0.34% (vol/vol) Triton X-100, and 0.9 mM cholesterol at 25°C for 24 h. The reaction mixture was extracted twice with 1 volume of chloroform. The cholesterol oxidation product in the concentrated chloroform extract was purified by preparative thin-layer chromatography (TLC) using a 2-mm silica gel 60F254 preparative TLC plate (Merck Millipore) as described previously [19].

### Measurement of the NMR spectrum of cholesterol oxidation products

The sample was dissolved in CDCl_3_ to a concentration of about 5–20 mg/ml. ^1^H-NMR and ^13^C-NMR spectra (300 MHz for ^1^H and 75 MHz for ^13^C) were recorded at 20°C with an AL300 FT NMR spectrometer (JEOL, Tokyo, Japan). Tetramethylsilane was used as an internal standard for the spectra.

### Materials

Triton X-100 and Triton-405 were purchased from Sigma-Aldrich, St. Louis, MO, USA. Tween 20, sodium cholate, sodium dodecyl sarcosinate (sarcosyl), sodium dodecyl sarcosinate (SDS), and laurylbenzenesulfonate (LBS) were from FUJIFILM Wako Pure Chemical, Osaka, Japan; and sodium polyoxyethylene alkyl (C12-13) ether sulfate (Emal 20CM) was from Kao, Tokyo, Japan. The organic solvents used were of the highest grade.

## Results

### Cloning of the two cholesterol oxidase genes

On the basis of the amino acid sequence of *Chromobacterium* sp. DS-1 cholesterol oxidase (GenBank/EMBL/DDBJ accession no. BAG70948.1), BLAST analysis was performed to identify putative cholesterol oxidases. We found two genes (*choRI* and *choRII*) encoding cholesterol oxidases belonging to the vanillyl-alcohol oxidase (VAO) family in the genome sequence of *R*. *erythropolis* PR4. Sequence data showed that the open reading frames of *choRI* and *choRII* were 1,764 and 1,767 bp encoding proteins of 587 and 588 amino acids with estimated molecular masses of 63,323 and 63,728 Da, respectively.

**Fig 1:**
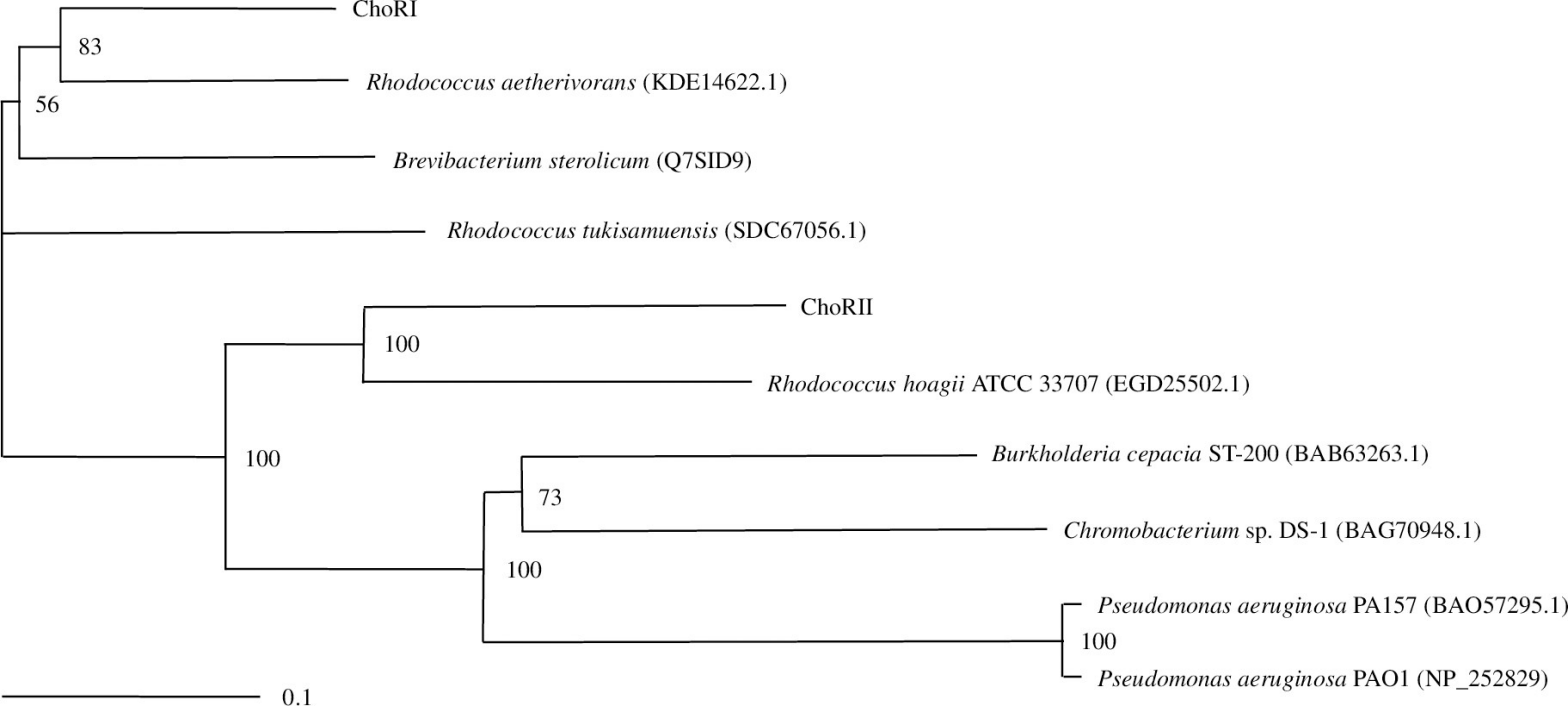
Phylogenetic tree of the cholesterol oxidases. The deduced amino acid sequences of ChoRI and ChoRII were compared to other homologous sequences. A phylogenetic tree was constructed based on the neighbor-joining method. The scale bar represents 0.1 substitutions per amino acid position. Bootstrap values greater than 50% are shown as percentages on branches. The GenBank/EMBL/DDBJ accession numbers are shown in parentheses.

Several amino acid sequences were found to exhibit significant similarities to ChoRI and ChoRII via a BLAST search. A phylogenetic tree was constructed by using amino acid sequences of ChoRI, ChoRII, and other bacterial cholesterol oxidases (Fig 1). The amino acid sequence of ChoRI showed significant similarity (57%) to that of ChoRII. ChoRI showed relatively high levels of similarity to the cholesterol oxidases from *R*. *aetherivorans* (78%), *B*. *sterolicum* (74%), and *R*. *tukisamuensis* (72%). Lower levels of similarity to ChoRI were found in cholesterol oxidases from *R*. *hoagie* (58%), *B*. *cepacia* ST-200 (49%), *Chromobacterium* sp. DS-1 (47%), and *P*. *aeruginosa* (45%). On the other hand, ChoRII showed high similarity to the enzyme from *R*. *hoagie* (69%) and relatively lower similarities to the enzymes from *R*. *aetherivorans* (58%), *R*. *tukisamuensis* (51%), *B*. *cepacia* ST-200 (51%), *Chromobacterium* sp. DS-1 (46%), *B*. *sterolicum* (45%), and *P*. *aeruginosa* (45%).

**Fig 2:**
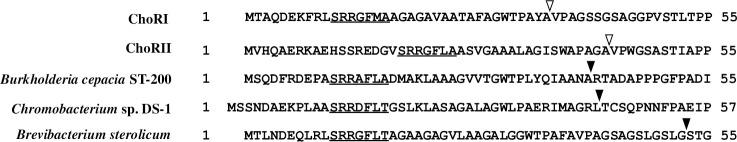
N-terminal amino acid sequences of VAO family cholesterol oxidases. These sequences include enzymes from ChoRI, ChoRII, *Burkholderia cepacia* ST-200 (accession no. BAB63263.1), *Chromobacterium* sp. DS-1 (no. BAG70948.1), and *Brevibacterium sterolicum* (no. Q7SID9). Putative consensus twin-arginine motifs are underlined. The open arrowheads indicate the presumptive cleavage sites of ChoRI and ChoRII. The filled arrowheads indicate the reported cleavage sites of the enzymes.

The twin arginine consensus motif of signal peptides is involved in Sec-independent translocation of proteins across the cytoplasmic membrane in bacteria [26]. The N-terminal amino acid sequences of ChoRI and ChoRII contained the distinctive twin-arginine motif-like signal sequences such as SRRGFMA between Ser-11 and Ala-17 for ChoRI and SRRGFLA between Ser-19 and Ala-25 for ChoRII (Fig 2). It was assumed that the signal peptidase recognition sequence (Ala-X-Ala) for ChoRI was AYA between Ala-34 and Ala-36, and that that for ChoRII was AGA between Ala-41 and Ala-43. Therefore, we speculated that the signal peptide of ChoRI seemed to be proteolytically processed between Ala-36 and Val-37, and that that of ChoRII might be processed between Ala-44 and Val-45 upon excretion and secretion in *R*. *erythropolis*. The predicted mature polypeptides of ChoRI and ChoRII consisted of 551 and 545 amino acid residues, with molecular masses of 59,590 and 59,369, respectively.

### Expression and purification of the recombinant cholesterol oxidases

**Fig 3:**
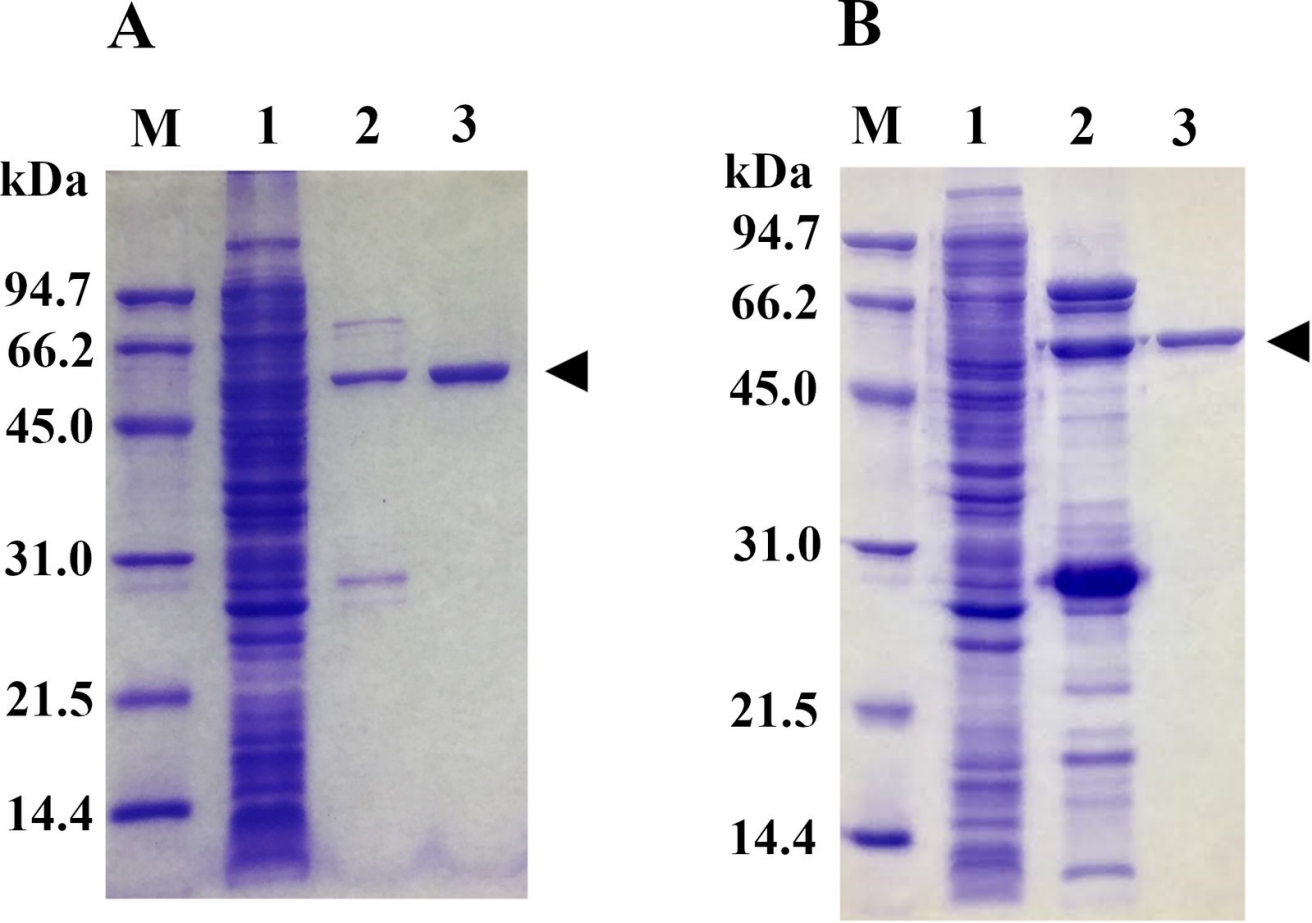
SDS-PAGE analysis of cholesterol oxidases during purification. Samples containing approximately 0.02 U of ChoRI (A) and ChoRII (B) were applied to SDS-12.5% (wt/vol) polyacrylamide gel. The gel was stained with CBB. Lane M, molecular size markers (kilodaltons); lane 1, cleared sonicated lysate; lane 2, fraction from the Ni-Sepharose column; lane 3, fraction from the DEAE-cellulose DE52 column. The gel was stained with Coomassie Brilliant Blue R-250 (CBB). The arrowhead indicates the size of the recombinant cholesterol oxidase.

The recombinant ChoRI and ChoRII were overexpressed by using the pET system. Table 1 summarizes the purification steps used. Since the purification step by Ni-affinity chromatography was incomplete in both cases, we used a DEAE-cellulose column to purify the enzymes further. In the end, ChoRI and ChoRII were purified 225- and 132-fold from the cell lysate, respectively. The specific activities of the purified cholesterol oxidase for cholesterol were and 8.7 and 0.40 U/mg of protein, respectively. The purified preparations showed single bands by SDS-PAGE (Fig 3). Both molecular masses were estimated to be approximately 59 kDa.

**Table 1:** Summary of the purification procedure for the recombinant cholesterol oxidases.

Enzyme	Step	Total	Total	Specific	Purifi-	Yield
		activity	protein	activity^a^	cation	
		(U)	(mg)	(U/mg)	(fold)	(%)
ChoRI	Cell lysate^b^	26.0	670	0.0388	1	100
	Ni-Sepharose	21.1	6.41	3.29	84.8	81.1
	DEAE-cellulose DE52	11.0	1.26	8.73	225	42.3
ChoRII	Cell lysate^b^	1.33	436	0.00304	1	100
	Ni-Sepharose	0.213	4.62	0.0461	15.2	16.0
	DEAE-cellulose DE52	0.0620	1.55	0.401	132	4.66

^a^Cholesterol oxidation activity was measured by following H_2_O_2_ generation.

^b^Cell lysate was obtained from 200 ml of a culture of *E*. *coli* Rosetta strain harboring the pET plasmid containing each cholesterol oxidase gene.

### Effect of temperature, pH, and chemicals on cholesterol oxidase activity

**Fig 4:**
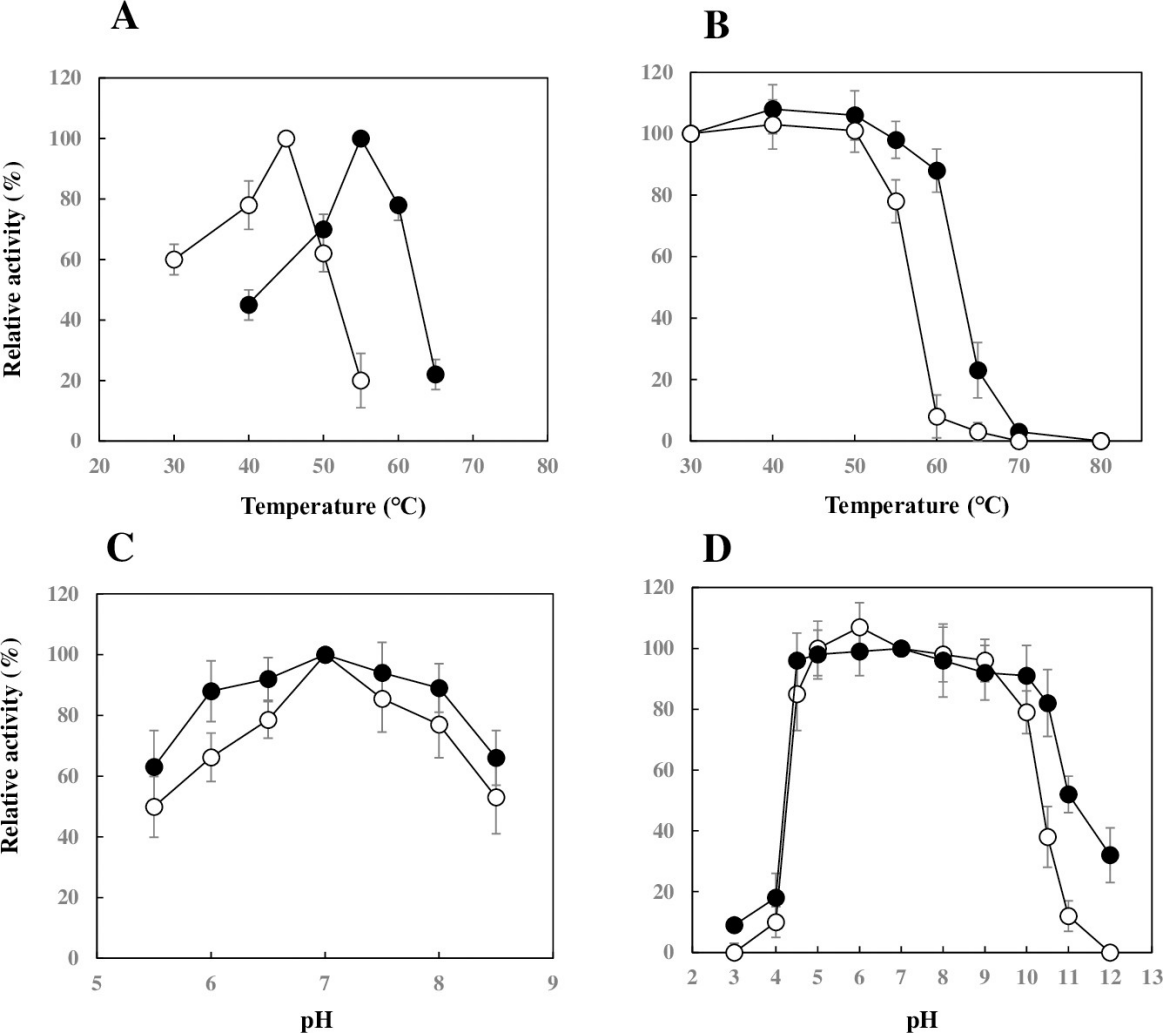
Effects of pH and temperature on the activity and stability of cholesterol oxidases. Effects of pH and temperature on the activity and stability of ChoRI (open circles) and ChoRII (closed circles) were measured. (A) Enzyme activity was determined by monitoring the A_249_ based on the λmax value of product formation at pH 7.0. (B) Thermal stability (closed circles) was assayed after incubation of the enzyme (0.2 U/ml) dissolved in 100 mM phosphate buffer (pH 7.0) for 30 min at the temperatures indicated, and the relative activity was examined by measuring H_2_O_2_ generation at 30°C. (C) Enzyme activity was assayed by following the consumption of oxygen at 30°C. (D) The pH stability was determined after incubation at 30°C for 1 h under the pH conditions indicated. The residual activity was examined by measuring H_2_O_2_ generation at 30°C. The buffer systems (100 mM) were glycine-HCl (pH 3.0), citrate-sodium citrate (pH 3.0–4.0), CH_3_COOH-CH_3_COONa (pH 5.0–5.5), NaH_2_PO_4_-Na_2_HPO_4_ (pH 5.5–7.5), Tris-HCl (pH 7.5–9.0), Na_2_CO_3_-NaHCO_3_ (pH 9.0–11.0), and NaCl-NaOH (pH 11.0–12.0). Data are the means of experiments performed in triplicate. Error bars represent the standard deviation.

The optimal temperatures and thermal stabilities of these two enzymes differed distinctively from each other (Fig 4A and 4B). ChoRI had an optimal temperature of 45°C (Fig 4A). By contrast, ChoRII showed an optimal temperature of 55°C. ChoRI was stable at temperatures from 30°C to 50°C after incubation for 30 min (Fig 4B). On the other hand, ChoRII was stable at temperatures from 30°C to 60°C. Thus, ChoRII exhibited high stability at wider temperature ranges. The optimal pHs of ChoRI and ChoRII were similar to one another (Fig 4C). Although these enzymes exhibited an optimal pH of 7.0, ChoRII showed relatively high activity between pH 6 to 8. ChoRI was stable from pH 4.5 to 10 (Fig 4D). By contrast, ChoRII was stable from pH 4.5 to 10.5 and showed higher stability than ChoRI even at pH 11 to 12.

The effects of metal ions on the enzyme activities of ChoRI and ChoRII were determined by measuring enzyme activity at 30°C for 1 h in the presence of 1 mM metal ions (Table 2). The addition of Ca^2+^, Mg^2+^, Fe^2+^, Ni^2+^, and Ag^+^ had no significant effect on the activities of these enzymes. The addition of Cu^2+^ and Mn^2+^ partially reduced the activity of ChoRII to 62% and 63%, respectively. ChoRI was relatively stable in the presence of Cu^2+^ and Mn^2+^. The activity levels of these enzymes were partially decreased in the presence of Zn^2+^. A chelating agent, EDTA, did not decrease the enzyme activity.

**Table 2:** Effects of metals and EDTA on the stability of the recombinant cholesterol oxidases.

Substrate	Relative^a^ activity (%)	
	ChoRI	ChoRII
None	100	100
Ca^2+^	104±6	86±2
Mg^2+^	104±10	105±2
Fe^2+^	101±10	105±9
Cu^2+^	88±2	62±7
Ni^2+^	100±3	110±2
Mn^2+^	100±7	63±4
Zn^2+^	73±10	79±8
Ag^+^	88±2	93±8
EDTA	113±5	129±3

^a^Enzyme activity that was measured by monitoring H_2_O_2_ generation is represented as a percentage of that obtained with cholesterol as the substrate. Mean values and standard deviation for three independent experiments are shown.

ChoRI and ChoRII were stable and partially or slightly activated in the presence of 0.5% (wt/vol) detergents including Tween 20, Triton X-100, Triton X-405, sodium cholate, sarcosyl, and Emal 20CM (Table 3). Both enzymes were completely inactivated by the addition of SDS and LBS. These enzymes were stable in the presence of 50% (vol/vol) hydrophobic organic solvents including toluene, *p*-xylene, and cyclooctane. However, the addition of methanol, ethanol, acetone, isopropanol, 1-butanol, and chloroform markedly inactivated the enzymes.

**Table 3:** Effects of detergents and organic solvents on the stability of the recombinant cholesterol oxidases.

Reagent group	Reagent^a^	Relative activity^b^	
		ChoRI	ChoRII
Detergent	None	100	100
	Tween 20	155±10	117±17
	Triton X-100	165±7	122±10
	Triton X-405	148±3	111±8
	Sodium cholate	174±3	128±8
	Sarcosyl	159±7	117±7
	Emal 20CM	175±4	110±10
	LBS	N.D.^c^	N.D. ^c^
	SDS	N.D. ^c^	N.D. ^c^
Organic solvent	None	100	100
	Methanol	N.D. ^c^	12±6
	Ethanol	N.D. ^c^	6±3
	Acetone	N.D. ^c^	N.D. ^c^
	Isopropanol	N.D. ^c^	7±3
	Butanol	N.D. ^c^	N.D. ^c^
	Chloroform	23±6	47±12
	Toluene	97±10	104±5
	*p*-Xylene	100±8	100±10
	Cyclooctane	107±6	114±14

^a^Sarcosyl, sodium dodecyl sarcosinate; Emal 20CM, sodium polyoxyethylene alkyl ether sulfate; SDS, sodium dodecyl sulfate.

^b^For the examination of enzymatic stability by the addition of detergents, cholesterol oxidase solution (0.2 U ml^-1^ in 50 mM sodium phosphate [pH 7.0]) containing 0.5% of each detergent was incubated at 30°C for 1 h. For the investigation of the stability in the presence of organic solvents, each organic solvent (1 ml) was added to 2 ml of the enzyme solution described above. The mixture was incubated at 37°C with shaking for 24 h. In both cases, the residual activity in the buffer was assayed by measuring the formation of H_2_O_2_. The relative residual activity was calculated as a percentage of the enzyme activity without each detergent or organic solvent. Mean values and standard deviations for three independent experiments are shown.

^c^N.D., not detected.

### Substrate specificity and enzyme kinetics

The substrate specificities of ChoRI and ChoRII differed significantly from one another (Table 4). ChoRI oxidized cholesterol, β-cholestanol, and β-sitosterol at high rates. The relative activities of ChoRI toward β-cholestanol and β-sitosterol were similar to those of cholesterol, but the activity levels toward ergosterol, pregnenolone, and epiandrosterone were markedly lowered. On the other hand, ChoRII exhibited 1.7- to 2.4-fold higher activity levels toward β-cholestanol, β-sitosterol, and stigmasterol than cholesterol. Slight activity was observed with epiandrosterone. In both enzymes, no activity was observed for dehydroepiandrosterone.

**Table 4:** Substrate specificity of cholesterol oxidase.

Substrate	Systematic name	Relative^a^ activity (%)	
		ChoRI	ChoRII
Cholesterol	Cholest-5-en-3β-ol	100	100
β-Cholestanol	5α-Cholestan-5-en-3β-ol	112±10	235±12
β-Sitosterol	Sitost-5-en-3β-ol	106±8	210±13
β-Stigmasterol	Stigmast-5-en-3β-ol	89±6	171±9
Ergosterol	Ergosta-5,7,22-trien-3β-ol	57±3	82±4
Pregnenolone	3β-Hydroxypregn-5-en-20-one	32±2	96±5
Epiandrosterone	5α-Androstan-3β-ol-17-one	3±1	29±6
Dehydroepiandrosterone	3β-Hydroxyandrost-5-en-17-one	N.D.	N.D.

^a^Enzyme activity, measured by monitoring H_2_O_2_ generation, is represented as a percentage of that obtained with cholesterol as the substrate. Mean values and standard deviation for three independent experiments are shown.

The Km and Vmax values of ChoRI and ChoRII for cholesterol were calculated by nonlinear regression using GraphPad Prism8 (Table 5). The Km values of ChoRI (22.6 μM) and ChoRII (22.1 μM) were similar to one another and were 0.24-, 0.29-, and 0.07-fold lower than that of *P*. *aeruginosa* PAO157 [18], *B*. *cepacia* ST-200 [15], and *Streptomyces* sp. SA-COO [15], respectively. On the other hand, the Vmax value of ChoRI (6.4 μmol/min/mg of protein) was higher than that of ChoRII (0.57 μmol/min/mg of protein). Thus, the catalytic efficiency (kcat/Km) of ChoRI is higher than that of ChoRII.

**Table 5:** Michaelis constant and maximum velocity of cholesterol oxidases.

Source	Km^a^	Vmax^a^	kcat^a^	kcat Km^-1^	Reference
	(μM)	(μmol min^-1^mg^-1^)	(s^-1^)	(s^-1^ μM^-1^)	
ChoRI	22.6±0.5	6.4±0.3	6.8±0.3	0.30±0.01	This study
ChoRII	22.1±0.3	0.57±0.10	0.61±0.11	0.03±0.01	This study
*Pseudomonas aeruginosa* PAO157	92.6±3.0	15.9±0.3	17.3±0.4	0.18±0.02	[18]
*Chromobacterium sp*. DS-1	26.2±0.9	10.4±0.7	10.9±0.7	0.42±0.03	[15]
*Burkholderia cepacia* ST-200	76.8±1.6	7.0±0.8	7.4±0.9	0.10±0.01	[15]
*Streptomyces* sp. SA-COO	315±17	3.9±0.9	3.8±0.9	0.01±0.00	[15]

^a^Cholesterol oxidation activity was assayed by measuring H_2_O_2_ generation. The Km and Vmax values for ChoRI and ChoRII were calculated by nonlinear regression using GraphPad Prism8. The data was obtained with the assay solution containing 0 to 1 mM cholesterol.

### Identification of the cholesterol oxidation product

ChoRI and ChoRII each oxidized cholesterol to produce one major product. These products were recovered as described previously [20]. They were structurally analyzed by ^1^H-NMR spectrometry. The chemical shift values of signals in ^1^H-NMR spectra of the product from ChoRI were as follows: 0.71 (3 H, s, 18-Me), 0.86 (3 H, d, *J* 6.60 Hz, 26-Me or 27-Me), 0.87 (3 H, d, *J* 6.60 Hz, 26-Me or 27-Me), 0.91 (3 H, d, *J* 6.60 Hz, 21-Me), 5.73 (1 H, s, 4-H). The ^1^H-NMR spectra of the product from ChoRI were identical to those previously reported for CEO [19]. On the other hand, the chemical shift values of signals in the ^1^H-NMR spectra of the product from ChoRII were as follows: 0.71 (3 H, s, 18-Me), 0.86 (3 H, d, *J* 6.60 Hz, 26-Me or 27-Me), 0.87 (3 H, d, *J* 6.60 Hz, 26-Me or 27-Me), 0.91 (3 H, d, *J* 6.60 Hz, 21-Me), 4.42 (1 H, dd, *J* 3.90 and 2.10 Hz, 6-H), 5.89 (1 H, d, *J* 0.60 Hz, 4-H), and 8.26 (1H, br, 6β-OOH). The ^1^H-NMR spectra of the product from ChoRII were coincident with those reported for HCEO [29, 30]. Furthermore, the identification of the products was confirmed by ^13^C-NMR spectrometry (Table 6). The chemical shift values of signals in the ^13^C-NMR of the products from ChoRI and ChoRII were identical to those reported for CEO and HCEO, respectively [27, 28].

**Table 6:** Assignments of ^13^C-NMR chemical-shift values^a^.

Position	Product from ChoRI	Product from ChoRII
1	35.74	35.48
2	33.98	34.19
3	199.56	199.46
4	123.73	129.12
5	171.59	170.17
6	32.95	86.00
7	32.07	33.98
8	35.65	34.10
9	53.84	53.46
10	38.61	37.92
11	21.06	20.82
12	39.52	39.41
13	42.41	42.46
14	55.90	56.03
15	24.18	24.04
16	28.17	28.12
17	56.15	59.96
18	11.96	11.91
19	17.40	18.69
20	35.71	35.97
21	18.65	18.67
22	36.17	36.06
23	23.83	23.74
24	39.68	39.57
25	28.00	28.01
26	22.54	22.53
27	22.79	22.81

^a^Spectra were taken at 75 MHz in CDCl_3_. Chemical shift values are expressed in ppm from an internal tetramethylsilane.

## Discussion

The genome sequence data of *R*. *erythropolis* PR4 showed that this strain possesses two VAO family cholesterol oxidases. The two genes (*choRI* and *choRII*) were cloned and expressed in *E*. *coli*. The similarity between the amino acid sequences of ChoRI and ChoRII was 57%. ChoRI and ChoRII showed significant similarities to the cholesterol oxidases from actinomycetes (Fig 1). Both ChoRI and ChoRII displayed lower similarities to the enzymes from beta- and gamma-proteobacteria such as *B*. *cepacia* ST-200, *Chromobacterium* sp. DS-1, and *P*. *aeruginosa* PA157. Among VAO family cholesterol oxidases, enzymes from *Chromobacterium* sp. DS-1, *B*. *cepacia* ST-200, *P*. *aeruginosa* PA157, and *B*. *sterolicum* have been characterized [15–20]. In particular, cholesterol oxidase from *Chromobacterium* sp. DS-1 exhibits higher stability than the other cholesterol oxidases at high temperatures and in the presence of detergents and organic solvents [19].

Three-dimensional structures of VAO family cholesterol oxidases such as *B*. *sterolicum* and *Chromobacterium* sp. DS-1 have been reported [29, 30]. The structure of *B*. *sterolicum* oxidase indicated that coenzyme FAD is covalently bound to His121. In addition, Glu475 and Arg477 play crucial roles in catalysis. Comparative amino acid sequence analysis showed that the amino acid residues His-121, Glu-475, and Arg-477 of the *B*. *sterolicum* oxidase were conserved in the sequence of ChoRI as the corresponding amino acid residues His-113, Glu-448, and Arg-450, and in the sequence of ChoRII as the corresponding residues His-113, Glu-449, and Arg-451, respectively.

The twin-arginine translocation (Tat) pathway is responsible for the transport of folded proteins across the cytoplasmic membrane by using a transmembrane proton electrochemical gradient [26]. Tat signal peptides each contain a consensus motif (S/TRRxFLK) within the Tat signal sequence, which is essential for the secretion of proteins via this system. These consensus motif-like sequences were also found in the signal sequences of cholesterol oxidases from ChoRI and ChoRII (Fig 2).

ChoRI and ChoRII showed thermal stability up to 50°C and 60°C after incubation for 30 min, respectively (Fig 4). The thermal stabilities of ChoRI and ChoRII were lower than those of VAO family cholesterol oxidases from *Chromobacterium* sp. DS-1, *B*. *cepacia* ST-200, and *P*. *aeruginosa* PA157 because these enzymes retained most of their activity even at 70°C after 30 min [16, 18, 19]. Detergents or organic solvents are often added to the reaction solution to dissolve water-immiscible cholesterol, although these reagents often inactivate cholesterol oxidases. ChoRI oxidase was remarkably activated by the addition of Tween 20, Triton X-100, Triton X-405, sodium cholate, sarcosyl, and Emal 20CM (Table 3). Such an activation of cholesterol oxidase by the addition of detergents was also found in *P*. *aeruginosa* PA157 cholesterol oxidase [18]. In terms of stability in organic solvents, ChoRI and ChoRII were stable in the presence of hydrophobic solvents including cyclooctane, *p*-xylene, and toluene. In contrast, these enzymes were highly sensitive to hydrophilic solvents such as methanol, ethanol, acetone, and isopropanol. Cholesterol oxidases from *Chromobacterium* sp. DS-1, *B*. *cepacia* ST-200, and *P*. *aeruginosa* PA157 were relatively stable in the presence of these hydrophilic solvents [16, 18, 19].

In terms of the substrate specificities, ChoRI catalyzed the oxidation of cholesterol, β-cholestanol, and β-sitosterol at high rates, and the relative activities to these substrates were similar to each other (Table 4). By contrast, ChoRII oxidized β-cholestanol, β-sitosterol, and stigmasterol at 2.4-, 2.1-, and 1.7-fold higher rates than cholesterol, respectively. No cholesterol oxidases that exhibit higher activity toward these substrates than cholesterol have been reported so far [16, 17, 19, 20]. β-Cholestanol is a minor component in the human body and in foods, but an increase in β-cholestanol concentration in serum has been reported to induce an autosomal recessive lipid-storage disorder named cerebrotendinous xanthomatosis (CTX) [31]. β-Sitosterol and stigmasterol are the most abundant plant sterols. *Rhodococcus* species are known for their ability to degrade phytosterols [32]. Since several bacterial cholesterol oxidases are implicated in the first step of cholesterol degradation [11], ChoRII might be involved in the initial step of phytosterol degradation. The Km values of ChoRI and ChoRII were relatively low among various cholesterol oxidases (Table 5). Thus, it was found that these enzymes show relatively high affinity toward cholesterol. The catalytic efficiency (kcat/Km) of ChoRI is higher than those of other oxidases except that of *Chromobacterium* sp. DS-1.

It has been known that cholesterol oxidases generally oxidize cholesterol to cholest-4-en-3-one. However, several VAO family cholesterol oxidases from *B*. *cepacia* ST-200, *Chromobacterium* sp. DS-1, and *P*. *aeruginosa* PA157 oxidize cholesterol to HCEO [16, 18, 19]. Our results showed that ChoRI and ChoRII produce CEO and HCEO from cholesterol, respectively. It is interesting that strain PR4 possesses two VAO family cholesterol oxidases that produce different cholesterol oxidation products.

In this study, we found that *R*. *erythropolis* PR4 has two VAO family cholesterol oxidases whose features differ distinctively from one another. In particular, we discovered that ChoRII preferentially oxidizes β-cholestanol and phytosterols (β-sitosterol and stigmasterol) rather than cholesterol. Further study of ChoRII might lead to the development of useful enzymes that can be employed to determine β-cholestanol levels for CTX diagnosis and to determine phytosterol levels in plants and foods.

## Supporting information

S1 Raw images (TIF)
