# Supplementary figures and images for: Cholesterol oxidase from Rhodococcus erythropolis with high specificity toward β-cholestanol and pytosterols
Source: PLoS One. 2020 Oct 26;15(10):e0241126. doi: 10.1371/journal.pone.0241126 (PMC7588053; doi:10.1371/journal.pone.0241126)

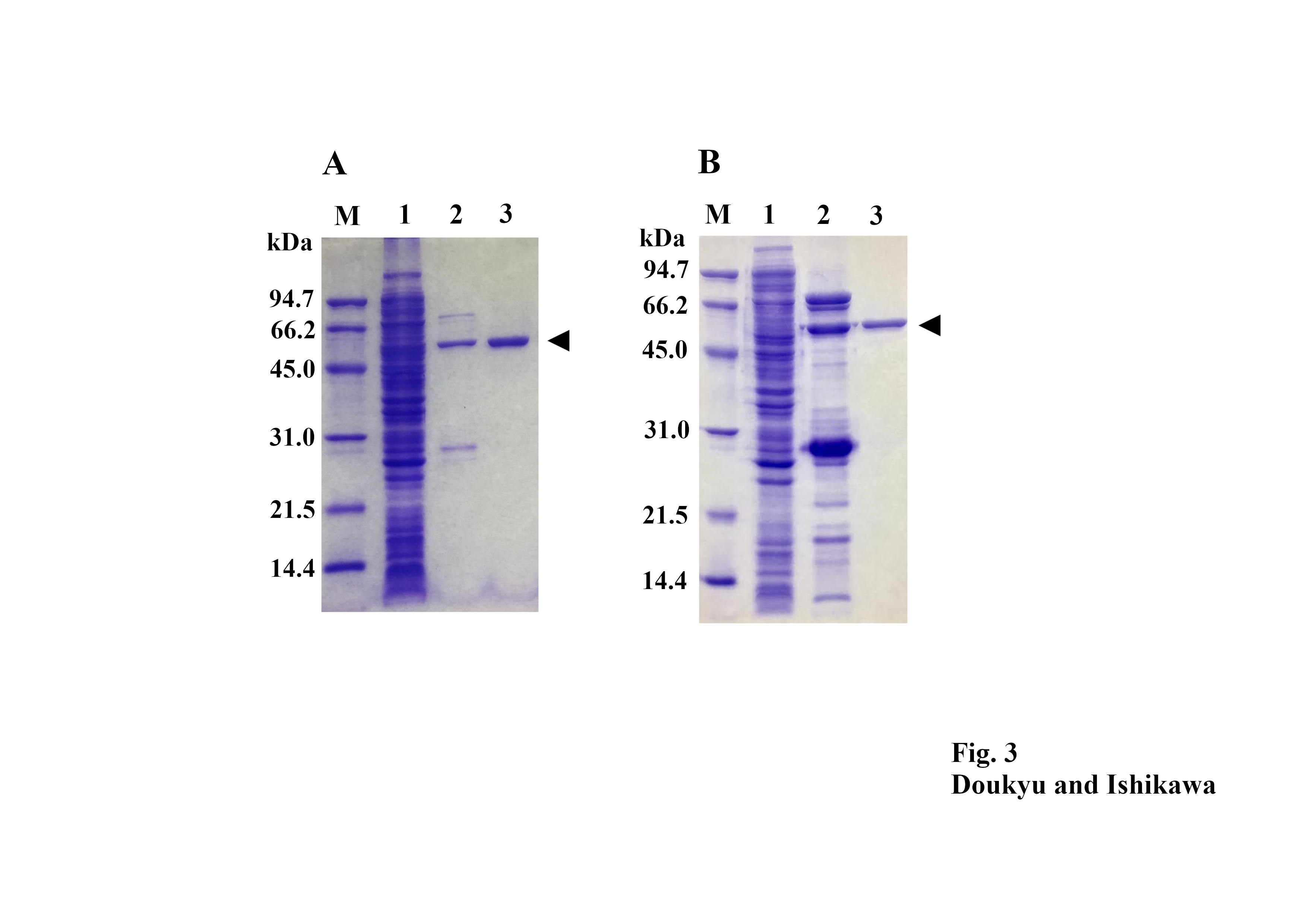

Supplement: S1 Raw images — (TIF) [file pone.0241126.s001.tif]
